# Synthesis of Novel Benzimidazole-Based Thiazole Derivatives as Multipotent Inhibitors of α-Amylase and α-Glucosidase: In Vitro Evaluation along with Molecular Docking Study

**DOI:** 10.3390/molecules27196457

**Published:** 2022-09-30

**Authors:** Rafaqat Hussain, Shahid Iqbal, Mazloom Shah, Wajid Rehman, Shoaib Khan, Liaqat Rasheed, Fazal Rahim, Ayed A. Dera, Sana Kehili, Eslam B. Elkaeed, Nasser S. Awwad, Majed A. Bajaber, Mohammed Issa Alahmdi, Hamad Alrbyawi, Hashem O. Alsaab

**Affiliations:** 1 Department of Chemistry, Hazara University, Mansehra 21300, Khyber Pakhtunkhwa, Pakistan; 2 Department of Chemistry, School of Natural Sciences (SNS), National University of Science and Technology (NUST), H-12, Islamabad 46000, Pakistan; 3 Department of Chemistry, Abbottabad University of Science and Technology (AUST), Abbottabad 22500, Pakistan; 4 Department of Clinical Laboratory Sciences, College of Applied Medical Sciences, King Khalid University, Abha 61413, Saudi Arabia; 5 Adham University College, Umm Al-Qura University, Makkah 21955, Saudi Arabia; 6 Department of Pharmaceutical Sciences, College of Pharmacy, AlMaarefa University, Riyadh 13713, Saudi Arabia; 7 Chemistry Department, Faculty of Science, King Khalid University, P.O. Box 9004, Abha 61413, Saudi Arabia; 8 Department of Chemistry, Faculty of Science, University of Tabuk, Tabuk 71491, Saudi Arabia; 9 Pharmaceutics and Pharmaceutical Technology Department, College of Pharmacy, Taibah University, Medina 42353, Saudi Arabia; 10 Department of Pharmaceutics and Pharmaceutical Technology, Taif University, P.O. Box 11099, Taif 21944, Saudi Arabia

**Keywords:** synthesis, benzimidazole bearing thiazole analogs, α-glucosidase, α-amylase, molecular docking

## Abstract

In this study, hybrid analogs of benzimidazole containing a thiazole moiety (**1**–**17**) were afforded and then tested for their ability to inhibit α-amylase and α-glucosidase when compared to acarbose as a standard drug. The recently available analogs showed a wide variety of inhibitory potentials that ranged between 1.31 ± 0.05 and 38.60 ± 0.70 µM (against α-amylase) and between 2.71 ± 0.10 and 42.31 ± 0.70 µM (against α-glucosidase) under the positive control of acarbose (IC_50_ = 10.30 ± 0.20 µM against α-amylase) (IC_50_ = 9.80 ± 0.20 µM against α-glucosidase). A structure–activity relationship (SAR) study was carried out for all analogs based on substitution patterns around both rings B and C respectively. It was concluded from the SAR study that analogs bearing either substituent(s) of smaller size (−F and Cl) or substituent(s) capable of forming hydrogen bonding (−OH) with the catalytic residues of targeted enzymes enhanced the inhibitory potentials. Therefore, analogs 2 (bearing meta-fluoro substitution), 3 (having para-fluoro substitution) and 4 (with *ortho*-fluoro group) showed enhanced potency when evaluated against standard acarbose drug with IC_50_ values of 4.10 ± 0.10, 1.30 ± 0.05 and 1.90 ± 0.10 (against α-amylase) and 5.60 ± 0.10, 2.70 ± 0.10 and 2.90 ± 0.10 µM (against α-glucosidase), correspondingly. On the other hand, analogs bearing substituent(s) of either a bulky nature (−Br) or that are incapable of forming hydrogen bonds (−CH_3_) were found to lower the inhibitory potentials. In order to investigate the binding sites for synthetic analogs and how they interact with the active areas of both targeted enzymes, molecular docking studies were also conducted on the potent analogs. The results showed that these analogs adopted many important interactions with the active areas of enzymes. The precise structure of the newly synthesized compounds was confirmed using several spectroscopic techniques as NMR and HREI-MS.

## 1. Introduction

Hyperglycemia, which can be characterized as a high blood glucose level, describes the metabolic disorder known as diabetes mellitus (DM), which is non-infectious and chronic. The alarming incidence of diabetes mellitus already includes 415 million cases worldwide [1], and that figure is expected to rise to 642 million by 2040 [2,3]. Carbohydratesare hydrolyzed by the enzymes glucosidase and amylase to produce blood glucose. Inhibitors such α-glucosidase, which hydrolyze oligosaccharides into simple sugar, are secreted by the pancreas and salivary glands to decrease the absorption of glucose from the small intestine. The inhibitors of α-amylase and α-glucosidase delay the absorption of glucose, which lowers postprandial blood glucose levels and is thought to be a therapeutic strategy for the treatment of diabetes. Diabetes has been treated using a variety of methods, but the most popular strategy relies on blocking α-glucosidase and α-amylase, the carbohydrase enzymes that delay glucose absorption [4,5]. The α-amylase initiates the hydrolysis of maltodextrins, starch and maltooligosaccharide, which in turn releases glucose molecules [6,7]. Similar to α-amylase, the α-glucosidase enzyme catalyzes the hydrolytic reaction that releases glucose molecules from the carbohydrates [8,9]. Type-2 diabetes (T2DM) risk is decreased by inhibitors of α-amylase and α-glucosidase including acarbose, voglibose and miglitol. Oral diabetes medications offer a quick onset of action and strong therapeutic effects, but they can also cause negative side effects. The primary disadvantage is their manner of action, which typically lessens diabetic symptoms rather treating the pathophysiology of the condition as a whole. Therefore, exploring active compounds with low risk of side effects is important for treating T2DM [10,11,12].

**Figure 1:**
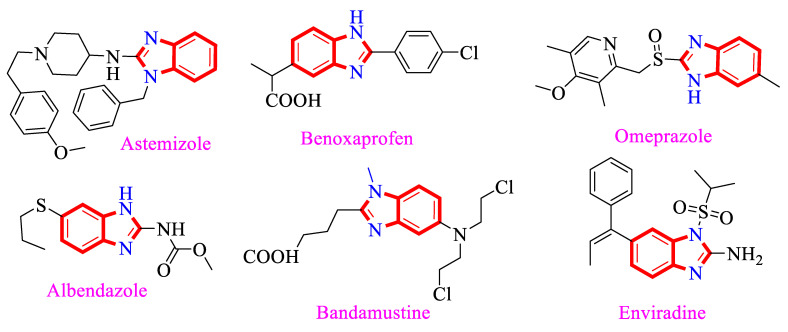
Benzimidazole skeleton containing drugs.

Proton pump inhibitors, anti-histaminic, anti-convulsant, anti-analgesic, anti-coagulant, anti-hypertensive, anti-cancer, anti-fungal and antiviral are only a few of the biological properties that benzimidazole and its derivatives are known to exhibit [13,14,15,16,17,18,19]. Some pharmacologically bioactive drugs, including those of albendazole, benoxaprofen, astemizole, enviradine, omeprazole and bendamastin, have a benzimidazole component in their structural framework (Figure 1) [20].

**Figure 2:**
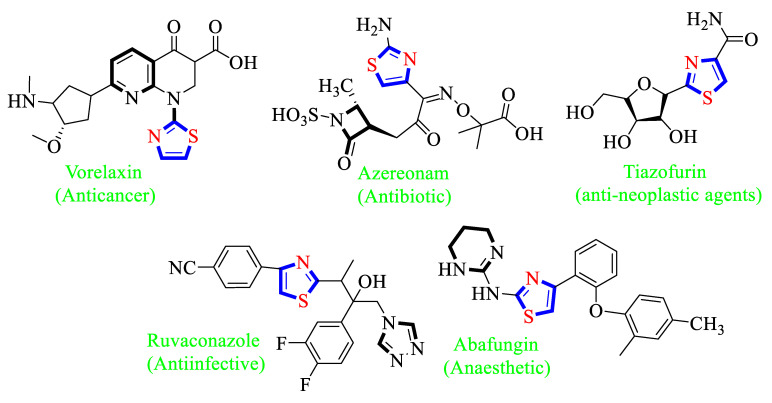
Biologically active drugs having a thiazole skeleton.

The thiazole motif bearing scaffolds displayed a diverse range of biological profile including anti-bacterial, anti-diuretic and anti-Alzheimer’s [21]. Additionally, thiazole-based hybrid scaffolds find applications as biologically interesting scaffolds and were known to have promising pharmacological significance such as anti-microbial [22], anti-inflammatory, analgesic [23], anti-hypoxic profile [24], anticancer [25], anti-hypertensive [26] and anti-asthmatic [27] effects. Moreover, it was noted that numerous commercially available biologically active drugs, such as abafungin, ruvaconazole, azereonam and vorelaxin contain thiazole skeleton in their structures due to the wide spectrum of the pharmacological activities of the thiazole ring (Figure 2) [28,29]. 

**Figure 3:**
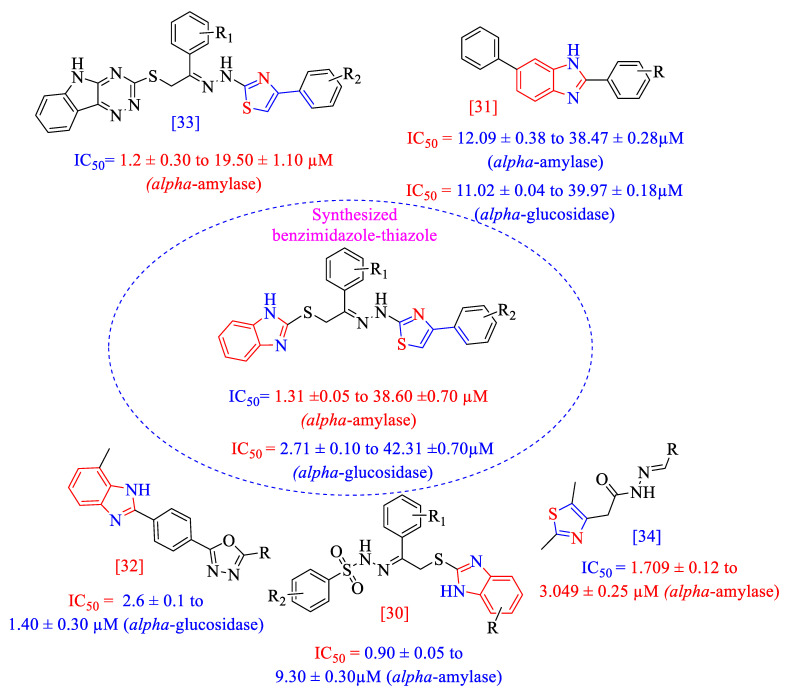
Rational of current study.

Keeping in mind the biological significance of previously reported benzimidazole [30,31,32] and thiazole [33,34] analogs as α-amylase and α-glucosidase inhibitors for the treatment of diabetic patients, benzimidazole and thiazole rings were combined in the same molecules to find lead molecules in an effort to further enhance the α-amylase and α-glucosidase inhibitory potentials (Figure 3). 

## 2. Results and Discussion

### 2.1. Chemistry

**Scheme 1:**
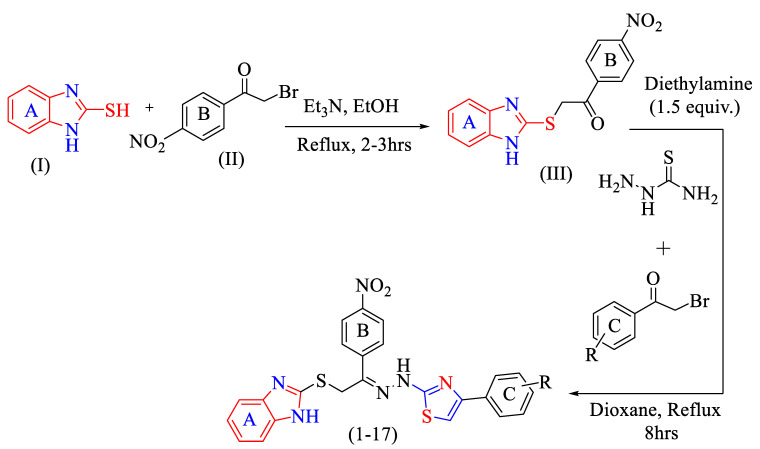
Preparation of hybrid analogs of thiazole incorporating a benzimidazole skeleton (**1**–**17**).

The preparation of benzimidazole-based thiazole derivatives (**1**–**17**) was completed in two steps. In the first step, 2-mercapto benzimidazole thiol (I) was treated with 4-nitro-substituted phenacyl bromide (II) in ethanol along with a catalytic amount of tri-ethyl amine under reflux condition to afford an intermediate (III) [35]. This intermediate was washed with petroleum ether in order to remove the impurities, and further this intermediate (III) was again reacted thiosemicarbazide and different substituted 2-bromoacetophenone via one-pot reaction in dioxane solvent and diethyl amine, and the subsequent residue was stirred under reflux for 8h to offer the synthesis of targeted benzimidazole-based thiazole derivatives (**1**–**17**). A thin-layer chromatographic (TLC) plate was used to monitor the conversion of reactants into products. All of the synthesized derivatives were purified by washing with petroleum ether and recrystallized from ethyl acetate to afford a purified form of the targeted benzimidazole-based thiazole analogs (**1**–**17**), which were then characterized through ^1^HNMR, ^13^CNMR and HREI-MS techniques (Scheme 1).

The precise structures of all newly afforded derivatives (**1**–**17**) were verified by NMR and HRMS. To better understand the spectral analysis of synthesized scaffolds (**1**–**17**), the synthesized compounds were divided into three major parts, including a benzimidazole part, a thiazole part and a 4-nitro benzene ring B and ring C respectively. The ^1^H-NMR results showed that protons of thiazole and benzimidazole (-NH) were resonated as a singlet at a chemical shift value of *δ*_H_ 7.28 and 12.55 ppm (for benzimidazole). In addition, a singlet was observed for the -NH proton present between benzimidazole and thiazole rings at *δ*_H_11.98ppm. Besides that, the remaining protons of benzimidazole were recorded as multiplets at approximately *δ*_H_ 7.43–7.12 ppm, and the characteristic -S-CH_2_- groups between the benzimidazole and thiazole heterocyclic rings were observed at *δ*_H_ 3.782 ppm asa singlet. The remaining protons of ring C were resonated at approximately *δ*_H_ 7.36–8.35 ppm as multiplets (*d, t, dt, dd*), depending on the attached electron-withdrawing (EW) or electron-donating (ED) groups. In the ^13^C-NMR spectra, the typical shifts near *δ*_13C_ 171.7 (C-thiazole), 155.6 (C=N), 150.2 (C-thiazole), 105.0 (CH-thiazole) and 37.5 (-S-CH_2_-) ppm reveal the presence of a thiazole ring, C=N and (-S-CH_2_-) moieties of the targeted benzimidazole-based thiazole analogs.

### 2.2. In Vitroα-Amylase and α-Glucosidase Inhibitory Activities *(**1**–**17**)*

**Figure 4:**
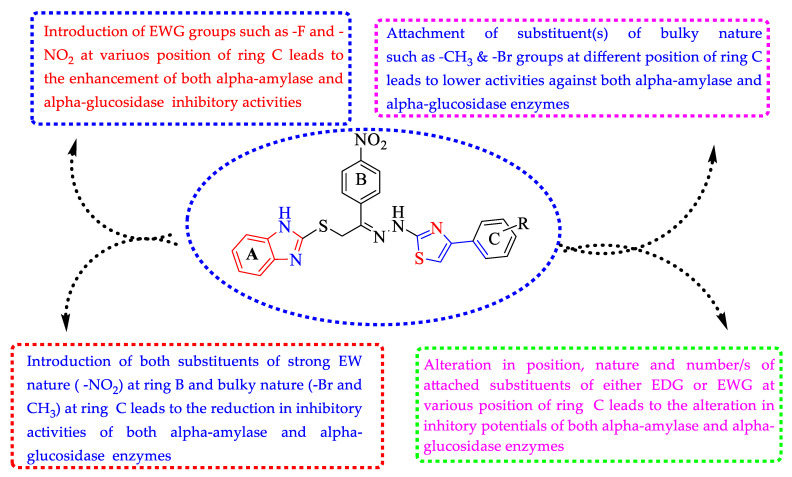
Summary of the structure–activity relationship (SAR) for inhibitory activities against α-amylase and α-glucosidase.

All of the newly afforded benzimidazole-based thiazole analogs were tested for inhibitory activities against α-amylase and α-glucosidase and were illustrated to have moderate to good inhibition properties, having IC_50_ values ranging between 1.30 ± 0.05 to 38.60 ± 0.70 µM (against α-amylase) and 2.70 ± 0.10 to 42.30 ± 0.70 µM (α-glucosidase) when compared to standard acarbose (IC_50_ = 10.30 ± 0.20 µM for α-amylase) (IC_50_ = 9.80 ± 0.20 µM for α-glucosidase) (Table 1). Based on substitutions pattern around aryl part C, SAR studies were conducted for all synthesized derivatives, and the obtained results showed that each part of the synthesized analogs including benzimidazole ring A, the thiazole moiety and ring B and ring C are actively contributing in the activity of α-glucosidase and α-amylase, and any variation found in potency was owed to varying number(s),positions, and EW or ED natures of substituent(s)that are attached to aryl part C, respectively (Figure 4). 

#### Structure–Activity Relationship (SAR) for Inhibitory Actions of α-Amylase and α-Glucosidase (**1**–**17**)

Analog 3 showed excellent potency among the synthesized series. By comparing analog 3bearing *ortho*-fluoro substitution on ring C along with a *para*-nitro group on ring B, with analogs 2 and 4 bearing a fluoro group at the *meta-* and *ortho*-positions of ring C; analog 3 displayed better activity toward α-amylase and α-glucosidase than its counterparts, 2 and 4. The difference in the inhibitory potentials of these three fluoro-substituted analogs toward α-amylase and α-glucosidase may be caused by different positions of the fluoro group around ring C (Table 1).

Analogs bearing chloro group(s) were found to display considerable inhibition against both these targeted enzymes (α-amylase and α-glucosidase). Among chloro-substituted analogs, analog 1 bearing di-Cl substitutions at the 3,4-position of the phenyl ring showed enhanced inhibitory potentials against both α-amylase and α-glucosidase enzymes when compared to other analogs, 14 (having a *para*-chloro group), 15 (bearing an *ortho*-chloro group) and 16 (with a *meta*-chloro group) bearing only one chloro group. This higher inhibitory potential of analog 1 might be due to greater numbers of attached chloro groups in comparison to other analogs 14, 15 and 16. However, analog 15 (IC_50_ = 5.20 ± 0.10 µM) bearing *ortho*-chloro substitution on the phenyl ring showed a superior activity for both alpha-amylase and alpha-glucosidase enzymes than its structurally similar counterparts 14 (IC_50_ = 16.40 ± 0.30 µM) and 16 (IC_50_ = 12.50 ± 0.20µM), demonstrating that altering the location of the substituent(s) around the phenyl ring has a significant impact on the inhibitory potentials (Table 1).

By comparing analog 10, bearing ortho-nitro substitution on phenyl ring C, with analogs 9 (having *para*-nitro substitution on ring C) and 17 (bearing *meta*-nitro substitution on ring C), analog 10exhibited better inhibitory potential than its structurally similar analogs 9 and 17. This suggests that the inhibitory potentials are increased by the nitro group’s ortho-position, which is more efficient for interactions with the active sites of both amylase and glucosidase enzymes. By moving the ortho-nitro group of ring C to its para-position, like in the case of analog 9, the inhibitory potential of analog 10 was drastically reduced. The potency was further decreased by shifting the *ortho*-nitro group to the *meta*-position as in analog 17. This difference in potency of these nitro-substituted analogs might be due to a different position of the nitro group around ring C (Table 1). 

It was noteworthy that the attachment of substituent(s) of either a bulky nature (–Br group) or substituent(s) incapable of interactions through hydrogen bonding (-CH_3_ group) at various position of ring C along with the nitro-substitution at the 4-position of ring B resulted in decreased inhibitory potentials against both α-amylase and α-glucosidase. Therefore, analogs 6 (bearing *para*-bromo on ring C), 7 (having *meta*-bromo on ring C) and 8 (with an *ortho*-bromo moiety on ring C) showed many-fold less potency when compared to either chloro-substituted analogs 16 (*meta*-chloro-substitution on ring C), 14 (having *para*-chloro-substitution on ring C) and 15 (with *ortho*-chloro-substituted ring C) or fluoro-substituted analogs 2 (*meta*-fluoro-substitution on ring C), 3 (having *para*-fluoro-substituted on ring C) and 4 (with *ortho*-fluoro-substitution on ring C) respectively (Table 1).

On the basis of aforementioned observation, it was concluded that analogs bearing substituent(s) of smaller size was found to be a better competitor of both targeted α-amylase and α-glucosidase, compared to analogs that bear substituent(s) of larger size. Moreover, it was also noted that inhibition properties for both α-amylase and α-glucosidase enzymes were greatly influenced by varying the number(s), positions and natures (electron-donating or electron-withdrawing groups) of substituent(s) around both rings B and C, respectively.

### 2.3. Molecular Docking Study

Understanding how synthetic analogs interact with enzymes (both α-amylase and α-glucosidase) was the main goal of the molecular docking study. After using a command prompt, a docking procedure was completed, and nine different poses of each ligand was obtained in a log file in which the top-ranked conformations (having the lowest binding affinity) were selected in order to conduct a more complete visualization of protein–ligand interactions (PLI).

To explore the binding modalities of the ligand with the active site of the protein, a molecular docking study was carried out (Table 2). Varied software has been used to achieve the significant results; these software were Auto Dock Vina and discovery studio visualizer (DSV) [36,37,38,39]. α-Amylase and α-glucosidase protein were retrieved from an online source (https://www.rcsb.org/ (18 May 2022)). α-Amylase and α-glucosidase protein 1b2y and 3w37, respectively, were downloaded in PDB format. A different step procedure was adopted for the exploration of protein–ligand interactions (PLI). In the first step, the retrieved protein was opened in Auto Dock Vina; the water molecule was removed, and, in addition, polar hydrogen and Kollman and Gasteiger charges were added. This was followed by the addition of a ligand molecule wherein charges added, and coordinates for configuration (X, Y and Z) and the dimension of these coordinates were 80 and grid box center X = 25.555Å, Y = 61.538Å and Z = 51.515Å with exhaustiveness = 8. The saved file in text format and both protein and ligand were also saved in PDBQT format. 1b2y (chain-A, **resolution** = 3.20 Å, native ligand pyroglutamic acid and residue == 496 amino acid) and 3w37 (chain-A, **resolution** = 1.70 Å, native ligand acarbose and residue == 913 amino acid).

**Figure 5:**
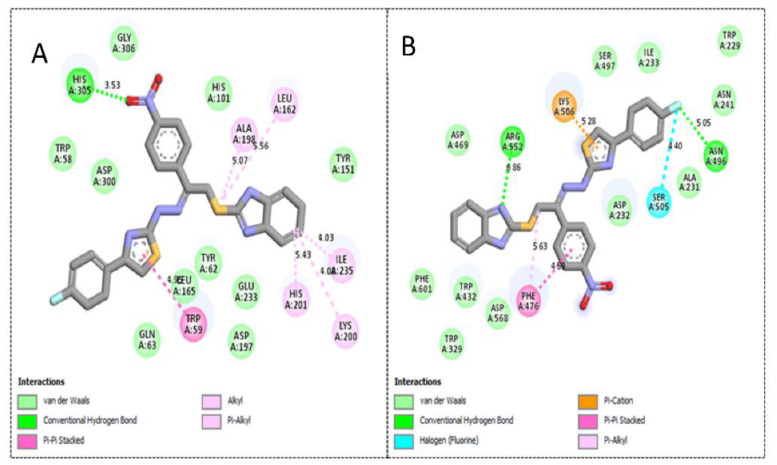
Representation of the protein–ligand interaction of compound **3.** (**A**) For compound **3** against α-amylase, while (**B**) is for the same compound **3** against α-glucosidase.

**Figure 6:**
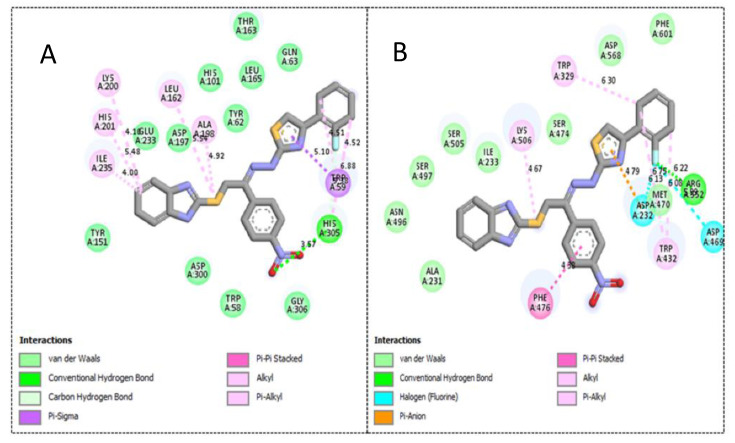
Representation of the protein–ligand interaction of compound **4.** (**A**) For compound **4** against α-amylase, while (**B**) is for the same compound **4** against α-glucosidase.

**Figure 7:**
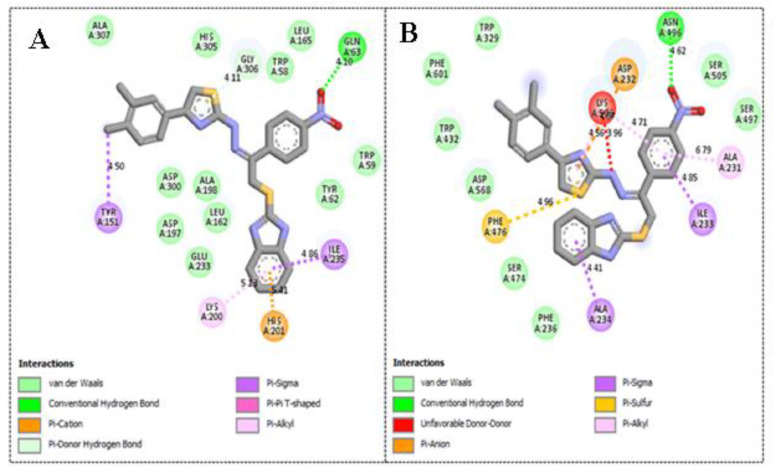
Representation of the protein–ligand interaction of compound **1.** (**A**) For compound **1** against α-amylase, while (**B**) is for the same compound **1** against α-glucosidase.

Interactions were discovered by using a command prompt in which the location of the target file was mentioned, and then processes were carried out. A total of nine poses for each ligand were obtained, which were explored in DSV to visualize binding interactions. A protein–ligand interaction (PLI) profile was summarized in (Figure 5, Figure 6 and Figure 7). 

## 3. Experimental

### 3.1. General Information

The Bruker AM 500 MHz machine NMR was used for the characterization of new compounds, and all necessary chemicals and reagents were bought from Sigma Aldrich, St. Louis, MO, USA. The splitted pattern of the peak was recorded as follows: dt, doublet of triplets, dd, doublet of doublets; sextet; sext, quintet; quint, q, quartet; t, triplet; d, doublet; m, multiplet, s, singlet. The coupling constant (J) was measured in hertz (Hz). High-resolution electron impact mass spectra (HREI-MS) were recorded on a Finnigan MAT-311A mass spectrometer (Germany). On precoated silica gel aluminium plates, thin-layer chromatography (TLC) was carried out (Kieselgel 60254, E. Merck, Germany). TLC plates were visualized by a UV lamp with a wavelength of 254 and 365 nm; the melting point was recorded with a Buchi M-560.

### 3.2. General Method for the Production of Thiazole Scaffolds Based on Benzimidazole *(**1**–**17**)*

Initially, benzimidazole-2-thiol I (1 equivalent) was reacted and stirred with 4-nitro-substituted phenacyl bromide II (1 equivalent) in ethanol (10mL) and Et_3_N (a few drops) to afford the formation of substrate III [35]. In the next step, substrate III (1 equivalent), thiosemicarbazide (1 equivalent) and different substituted phenacyl bromide (1 equivalent) were reacted and refluxed in dioxane (10mL) and diethyl amine (1.5 equivalent) via a one-pot reaction. The residue was stirred for 8hrs under reflux. The solvent was evaporated on the completion of the reaction by employing reduced pressure to give a solid residue, which was further washed with petroleum ether and then recrystallized with ethyl acetate to access the formation of targeted benzimidazole-based thiazole derivatives (**1**–**17**) in appropriate yield. The precise structures of all of the newly synthesized derivatives were confirmed by using NMR and HREI-MS spectroscopic methods. 

Appendix: 3.2. α-amylase inhibition assay, 3.2. α-glucosidase inhibition assay, 3.4. Doc king protocol [32] and 3.5. General procedures along with spectral analysis are provided in Supplementary Materials [40,41,42].

## 4. Conclusions

In conclusion, by employing acarbose as a standard drug, an approach was established for the synthesis of hybrid analogs of benzimidazole containing thiazole (**1**–**17**), which were then tested for their inhibition properties against α-amylase and α-glucosidase. All of the synthesized analogs were found to display a varied range of inhibition properties against both enzymes, with IC_50_ values of 1.31 ± 0.05 to 38.60 ± 0.70 µM (for α-amylase) and 2.71 ± 0.10 to 42.31 ± 0.70 µM (for α-glucosidase) when compared to standard acarbose (IC_50_ = 10.30 ± 0.20 µM for α-amylase) (IC_50_ = 9.80 ± 0.20µM for α-glucosidase). Among the synthesized series, seven analogs such as 1, 2, 3, 4, 5, 10 and 15 were found to be more potent than standard acarbose, with IC_50_ values of 2.20 ± 0.10, 4.10 ± 0.10, 1.30 ± 0.05, 1.90 ± 0.10, 3.60 ± 0.20, 7.20 ± 0.20 and 5.20 ± 0.10 (against α-amylase) and 3.90 ± 0.20, 5.60 ± 0.10, 2.70 ± 0.10, 2.90 ± 0.10, 4.30 ± 0.30, 9.60 ± 0.20 and 6.30 ± 0.10, respectively (against α-glucosidase). Besides that, the remaining ten analogs also exhibited considerable inhibitory potentials but were found to be less potent than standard acarbose. For active analogs, molecular docking was devised in order to examine the binding locations of synthetic analogs and how they interact with the catalytic cavity of amino acids in enzymes. The findings showed that these analogs adopted a number of significant interactions with the active regions of enzymes. In addition, various spectroscopic tools such as HREI-MS and NMR were employed to confirm the precise structure of the synthesized compounds. 

## Figures and Tables

**Table 1:** The α-amylase and α-glucosidase inhibition properties (in vitro) by benzimidazole-containing thiazole analogs along with different substituent(s) (**1**–**17**).

Synthesized Compounds	R	α-Amylase IC_50_ [µM]	α-Glucosidase IC_50_ [µM]
**1**	3,4-dichloro	2.20 ± 0.10	3.90 ± 0.20
**2**	3-fluoro	4.10 ± 0.10	5.60 ± 0.10
**3**	4-fluoro	1.30 ± 0.05	2.70 ± 0.10
**4**	2-fluoro	1.90 ± 0.10	2.90 ± 0.10
**5**	4-hydroxy	3.60 ± 0.20	4.30 ± 0.30
**6**	4-bromo	18.50 ± 0.30	25.80 ± 0.50
**7**	3-bromo	34.70 ± 0.70	36.40 ± 0.70
**8**	2-bromo	20.30 ± 0.50	29.30 ± 0.60
**9**	4-nitro	16.40 ± 0.30	19.60 ± 0.40
**10**	2-nitro	7.20 ± 0.20	9.60 ± 0.20
**11**	4-methyl	24.40 ± 0.30	25.90 ± 0.40
**12**	3-methyl	38.60 ± 0.70	34.40 ± 0.70
**13**	2-methyl	34.20 ± 0.60	37.50 ± 0.70
**14**	4-chloro	16.40 ± 0.30	17.30 ± 0.30
**15**	2-chloro	5.20 ± 0.10	6.30 ± 0.10
**16**	3-chloro	12.50 ± 0.20	13.60 ± 0.20
**17**	3-nitro	28.40 ± 0.40	29.60 ± 0.40
Standard acarbose drug		10.30 ± 0.20	9.80 ± 0.20

**Table 2:** The number(s) of hydrogen bond, IC_50_ values, binding energies, interacting residues and the closest residues of the selected docked analogs into the active site of both α-amylase and α-glucosidase enzymes.

Active Derivatives	Name of Enzyme	IC_50_ [µM]	Free Binding Energy (kcal/mol)	Number of HBs	Number of Closest Residues	Interacting Residues
**3**	α-amylase	1.30 ± 0.05	−12.13	1	17	His305, Trp59, Ala198, Leu162, Lys200, His201 and Ile235
	α-glucosidase	2.70 ± 0.10	−11.48	2	16	Phe476, Asp232, Trp432, Met470, Asp469, Arg552, Trp329 and Lys506
**4**	α-amylase	1.90 ± 0.10	−10.87	1	17	Trp59, His201, Lys200, Ile235, Leu162, Ala198 and His305
	α-glucosidase	2.90 ± 0.10	−10.19	1	16	Phe476, Ser505, Asn496, Lys506 and Arg552
**1**	α-amylase	2.20 ± 0.10	−9.23	1	17	Tyr151, Lys200, His201, Ile235 and Gln63
	α-glucosidase	3.90 ± 0.20	−8.98	1	15	Phe476, Ala 234, Ile233, Ala231, Asn496, Asp232 and Lys506

## Data Availability

The data presented in this study are available in Supplementary Materials.

## Supplementary Materials

The following supporting information can be downloaded at: www.mdpi.com/article/10.3390/molecules27196457/s1, Supplementary Information file [40,41,42].

## Sample Availability

Samples of the compounds are not available from the authors.
