# Supplementary material for: Synthesis of Novel Benzimidazole-Based Thiazole Derivatives as Multipotent Inhibitors of α-Amylase and α-Glucosidase: In Vitro Evaluation along with Molecular Docking Study
Source: Molecules. 2022 Sep 30;27(19):6457. doi: 10.3390/molecules27196457 (PMC9572811; doi:10.3390/molecules27196457)
Supplement: Supplementary file 1 [file molecules-27-06457-s001.zip › molecules-1928525-supplementary.pdf]

Synthesis of novel benzimidazole-based thiazole derivatives as multipotent inhibitors of α-amylase and α-glucosidase: *In vitro* evaluation along with molecular docking study

Rafaqat Hussain[1], Shahid Iqbal[2]*, Mazloom Shah[3], Wajid Rehman[1]*, Shoaib Khan[1], Liaqat Rasheed[1], Fazal Rahim[1],Ayed A. Dera[4], Sana Kehili[5], Eslam B. Elkaeed[6], Nasser S. Awwad[7], Majed A. Bajaber[7], Mohammed Issa Alahmdi[8], Hamad Alrbyawi[9], Hashem O. Alsaab[10]

[1]Department of Chemistry, Hazara University, Mansehra-21300, Khyber Pakhtunkhwa, Pakistan

[2]Department of Chemistry, School of Natural Sciences (SNS), National University of Science and Technology (NUST), H-12, Islamabad, 46000, Pakistan

[3]Department of Chemistry, AbbottabadUniversity of Science and Technology (AUST) Abbottabad, Pakistan.

[4]Department of Clinical Laboratory Sciences, College of Applied Medical Sciences, King Khalid University, Abha, Saudi Arabia.

[5]Adham University College, Umm Al-Qura University, Makkah 21955, Saudi Arabia.

[6]Department of Pharmaceutical Sciences, College of Pharmacy, AlMaarefa University, Riyadh 13713, Saudi Arabia.

[7]Chemistry Department, Faculty of Science, King Khalid University, P.O. Box 9004, Abha 61413, Saudi Arabia.

[8]Department of Chemistry, Faculty of Science, University of Tabuk, Tabuk- 71491, Saudi Arabia.

[9]Pharmaceutics and Pharmaceutical Technology Department, College of Pharmacy, Taibah University, Medina 42353, Saudi Arabia

[10]Department of Pharmaceutics and Pharmaceutical Technology, Taif University, P.O. Box 11099, Taif 21944, Saudi Arabia.

***To whom corresponding should be addressed**

shahidgcs10@yahoo.com(Shahid Iqbal) and Wajid Rehman (sono_waj@gmail.com)

3.2. α-amylase inhibition assay

The a-amylase inhibition was determined by an assay modified from Kwon, Apostolidis& Shetty. Five hundred microlitersof plant extract and all reagents were modified to 40 lL in this assay. A total of 40 lL of sample and 40 lL of 0.02 M sodiumphosphate buffer (pH 6.9 with 0.006 M sodium chloride) containinga-amylase solution (Porcine pancreatic a-amylase) (0.5 mg/ml)were incubated at 25^0C for 10 min. After pre-incubation, 40 lLofa 1% starch solution in 0.02M sodium phosphate buffer (pH 6.9with 0.006 M sodium chloride) was added to each tube at 5 s intervals.The reaction mixtures were then incubated at 25 _C for 10 min.The reaction was stopped with 100 lL of dinitrosalicylic acid colorreagent. The test tubes were then incubated in a

boiling water bathfor 5 min and cooled to room temperature. The reaction mixturewas then diluted after adding 900 lL distilled water and the absorbancewas measured at 540 nm. Calculation of the concentration ofcompound can be finding with the help of equation given below.

3.3. α-glucosidase inhibition assay

The α-glucosidase inhibition activity was performed with slightmodifications as given by Rahim et al. Total volume of 100 µLreaction mixture contained, 70 µL 50mM phosphate buffer pH 6.8, 10µL (0.5mM in methanol) test compound, followed by the addition of 10µL (0.057 units, Sigma Inc.) enzyme solution in the buffer. The contentswere mixed, pre-incubated for 10 min at 37 °C and pre-read at 400 nm.The reaction was initiated by the addition of 10 µL of 0.5mM substrate(p-nitrophenyl glucopyranoside, Sigma Inc.). After 30 min of incubationat 37 °C, the absorbance of p-nitrophenol was measured at 400 nm usingthe Synergy HT 96-well plate reader, BioTek, USA. Acarbose was usedas positive control. All experiments were carried out in triplicates(mean ± SEM, n=3). Percent inhibition was calculated by the following equation:

Inhibition (%) = (Abs ofControl−Abs of Test/Abs of Control) × 100

Active compound solutions were suitably diluted, and their inhibitionstudies were determined. Data obtained was used for the determinationof IC50 values (concentration at which there is 50% enzymeinhibition) using EZ-Fit Enzyme Kinetics Software (PerrellaScientificInc. Amherst, USA).

3.4. Molecular Docking protocol

Molecular docking study was conducted by using AutoDock Vina. In this study the synthesized compounds were analyzed against α-amylase and α-glucosidase enzyme. The **pdbqt** files for protein and ligands preparation and grid box creation were completed using Graphical User Interface program AutoDock Tools (ADT). ADT assigned polar hydrogens, united atom Kollman charges, solvation parameters and fragmental volumes to the protein. AutoDock saved the prepared file in PDBQT format. AutoGrid was used for the preparation of the grid map using a grid box. The grid size was set to 80 × 80 × 80 xyz points with grid box center X = 25.555Å, Y = 61.538Å and Z = 51.515Å with exhaustiveness = 8. A scoring grid is calculated from the ligand structure to minimize the computation time. AutoDock/Vina was employed for docking

using protein and ligand information along with grid box properties in the configuration file. AutoDock/Vina employs iterated local search global optimizer [1, 2]. During the docking procedure, both the protein and ligands are considered as rigid. The results less than 1.0 Å in positional root-mean square deviation (RMSD) was clustered together and represented by the result with the most favorable free energy of binding. The pose with lowest energy of binding or binding affinity was extracted and aligned with receptor structure for further analysis [3].

3.5. Spectral analysis

3.5.1.(E)-2-(2-(2-((1H-benzo[d]imidazol-2-yl)thio)-1-(3,4-dichlorophenyl)ethylidene) hydrazinyl)-4-(3,4-dichlorophenyl)thiazole (1)

Yield: (68%); mp. 206-207 °C;^{1}HNMR: (500 MHz, DMSO-d_6) δ 12.55 (s, 1H, -NHbi), 11.98 (s, 1H, -NH), 8.35(d, $J_{(3,2)/(5,6)}$ = 7.8Hz, 2H, H-3/H-5, ArH), 8.07 (d, $J_{(2,3)/(6,5)}$ = 7.6Hz, 2H, H-2/H-6,ArH), 7.92 (d, $J_{(2',6')}$ = 1.7Hz, 1H, Ar'H), 7.86 (d, $J_{(6',5')}$ = 7.4 Hz,$J_{(6',2')}$ = 1.8Hz, 1H, Ar'H), 7.56 (d, $J_{(5',6')}$ = 7.6Hz, 1H, Ar'H), 7.43 (m, 2H, bi-H), 7.28 (s, 1H, thiazole-H), 7.12 (m, 2H, bi-H), 3.78 (s, 2H, -CH_2) ^{13}CNMR (125 MHz, DMSO-d_6): δ171.7, 155.6, 150.2, 150.2, 147.1, 140.1, 138.9, 138.9, 133.4, 132.7, 132.5, 130.7, 128.8, 127.7, 127.7, 127.0, 127.0, 123.0, 123.0, 115.2, 115.2, 105.0, 37.5: HREI-MS: m/z calcld for $C_{24}H_{16}Cl_2N_6O_2S_2[M]^+$ 554.0153 Found 554.0148.

3.5.2.(E)-2-(2-(2-((1H-benzo[d]imidazol-2-yl)thio)-1-(4-nitrophenyl)ethylidene)hydrazinyl)-4-(3-fluorophenyl)thiazole (2)

Yield: (78%); mp. 200-201 °C;^{1}HNMR: (500 MHz, DMSO-d_6) δ 12.55 (s, 1H, -NHbi), 11.98 (s, 1H, -NH), 8.35(d, $J_{(3,2)/(5,6)}$ = 7.8Hz, 2H, H-3/H-5, ArH), 8.07 (d, $J_{(2,3)/(6,5)}$ = 7.6Hz, 2H, H-2/H-6,ArH), 7.62 (m, 1H, Ar'H), 7.56 (m, 2H, Ar'H), 7.43 (m, 2H, bi-H), 7.28 (s, 1H, thiazole-H), 7.20 (m, 1H, Ar'H), 7.12 (m, 2H, bi-H), 3.78 (s, 2H, -CH_2).^{13}CNMR (125 MHz, DMSO-d_6): δ171.7, 155.6, 150.2, 150.2, 147.1, 140.1, 138.9, 138.9, 134.6, 127.7, 127.7, 127.5, 127.0, 127.0, 123.1, 123.0, 123.0,115.9, 115.5, 115.2, 115.2, 105.0, 37.5: HREI-MS: m/z calcld for $C_{24}H_{17}FN_6O_2S_2[M]^+$ 504.0838 Found 504.0833.

3.5.3.(E)-2-(2-(2-((1H-benzo[d]imidazol-2-yl)thio)-1-(4-nitrophenyl)ethylidene)hydrazinyl)-4-(4-fluorophenyl)thiazole (3)

Yield: (70%); mp. 199-200 °C;^{1}HNMR: (500 MHz, DMSO-d_6) δ11.90 (s, 1H, -NHbi), 11.80 (s, 1H, -NH), 8.79 (d, $J_{(2, 3)/(6, 5)}$ = 6.7Hz, 2H, H-2/H-6, ArH), 8.52 (d, $J_{(3, 2)/(5, 6)}$ = 7.7Hz, 2H, H-3/H-5,ArH), 8.45 (s, 1H, thiazole-H), 7.58 (d, $J_{(2', 3')/(6', 5')}$ = 6.6Hz, 2H, H-2/H-6, Ar'H),7.38 (d, $J_{(3', 2')/(5', 6')}$ = 6.6Hz, 2H, H-3/H-5,Ar'H), 7.02 (d, J = 7.9Hz, 2H, bi-H), 6.61 (d, J = 7.3Hz, 2H, bi-H), 3.81 (s, 2H, -CH_2) ^{13}CNMR (125 MHz, DMSO-d_6): δ172.5, 168.9, 167.4, 152.9, 152.2, 148.4, 145.6, 141.9, 141.1, 139.5, 129.0, 128.7, 126.2, 125.9, 124.8, 123.0, 121.2, 117.7, 115.0, 110.6, 110.0, 109.2, 107.5, 39.4: HREI-MS: m/z calcld for $C_{24}H_{17}FN_6O_2S_2$ $[M]^+$ 504.0838 Found 504.0833.

3.5.4.(E)-2-(2-(2-((1H-benzo[d]imidazol-2-yl)thio)-1-(4-nitrophenyl)ethylidene)hydrazinyl)-4-(2-fluorophenyl)thiazole (4)

Yield: (75%); mp. 199-198 °C;^{1}HNMR: (500 MHz, DMSO-d_6) δ 12.55 (s, 1H, -NHbi), 11.98 (s, 1H, -NH), 8.35(d, $J_{(3, 2)/(5, 6)}$ = 7.8Hz, 2H, H-3/H-5, ArH), 8.07 (d, $J_{(2, 3)/(6, 5)}$ = 7.6Hz, 2H, H-2/H-6,ArH), 7.72-7.74 (m, 2H, Ar'H), 7.48 (d, $J_{(6', 5')}$ = 7.5 Hz,$J_{(6', 2')}$ = 1.7Hz, 1H, Ar'H), 7.44 (m, 2H, bi-H), 7.28 (s, 1H, thiazole-H), 7.25 (m, 1H, Ar'H), 7.12 (m, 2H, bi-H), 3.78 (s, 2H, -CH_2) ^{13}CNMR (125 MHz, DMSO-d_6): δ171.7,158.3, 155.6, 150.2, 147.8, 147.1, 140.1, 138.9, 138.9, 130.3, 129.1, 127.7, 127.7, 127.0, 127.0, 124.8, 123.5, 123.0, 123.0, 115.2, 115.2, 115.7, 105.0, 37.5: HREI-MS: m/z calcld for $C_{24}H_{16}Cl_2N_6O_2S_2$ $[M]^+$ 504.0838 Found 504.0833.

3.5.5.(E)-4-(2-(2-(2-((1H-benzo[d]imidazol-2-yl)thio)-1-(4-nitrophenyl)ethylidene)hydrazinyl) thiazol-4-yl)phenol (5)

Yield: (69%); mp. 198-197 °C;^{1}HNMR: (500 MHz, DMSO-d_6) δ 11.84 (s, 1H, NH), 11.62 (s, 1H, NH), 9.30 (s, 1H, OH), 8.78 (d, $J_{(3, 2)/(5, 6)}$=6.7Hz, 2H, H-3/H-5, ArH), 8.51(d, $J_{(2, 3)/(6, 5)}$ = 6.8Hz, H-2/H-6,ArH), 7.57 (d, , $J_{(3', 2')/(5', 6')}$ = 7.8Hz, H-3/H-5, Ar'H), 7.10 (d,$J_{(2', 3')/(6', 5')}$ = 6.7Hz, H-2/H-6,Ar'H), 6.86 (d, J = 6.7Hz, 2H, bi-H), 6.60 (d, J = 7.3Hz, 2H, bi-H), 3.84 (s, 2H, -CH_2), ^{13}CNMR (125 MHz, DMSO-d_6): δ168.4, 167.2, 148.7, 146.4, 142.1, 141.5, 137.4, 136.3, 134.5, 134.0, 133.4, 132.7, 132.3, 131.8, 131.0, 130.9, 129.4, 127.3, 126.3, 125.8, 120.7, 117.6, 116.0, 110.3, 40.0: HREI-MS: m/z calcld for $C_{24}H_{18}N_6O_3S_2$$[M]^+$ 502.0783 Found 502.0777.

3.5.6.(E)-2-(2-(2-((1H-benzo[d]imidazol-2-yl)thio)-1-(4-nitrophenyl)ethylidene)hydrazinyl)-4-(4-bromophenyl)thiazole (6)

Yield: (77%); mp. 210-209 °C;^{1}HNMR: (500 MHz, DMSO-d_6) δ 12.55 (s, 1H, -NHbi), 11.98 (s, 1H, -NH), 8.35(d, $J_{(3, 2)/(5, 6)}$ = 7.8Hz, 2H, H-3/H-5, ArH), 8.07 (d, $J_{(2, 3)/(6, 5)}$ = 7.6Hz, 2H, H-2/H-6,ArH), 7.76(d, $J_{(2', 3')/(6', 5')}$ = 7.9Hz, 2H, H-2/H-6, Ar'H), 7.56 (d, $J_{(3', 2')/(5', 6')}$ = 7.6Hz, 2H, H-3/H-5,Ar'H), 7.43 (m, 2H, bi-H), 7.28 (s, 1H, thiazole-H), 7.12(m, 2H, bi-H), 3.76 (s, 2H, -CH_2) ^{13}CNMR (125 MHz, DMSO-d_6): δ171.6, 155.6, 150.2, 150.2, 147.1, 140.1, 138.9, 138.9, 132.2, 132.2, 132.1, 128.3, 128.3, 127.2, 127.2, 127.0, 127.0, 123.0, 123.0, 115.2, 115.2, 105.0, 37.5: HREI-MS: m/z calcld for $C_{24}H_{17}BrN_6O_2S_2$ $[M]^+$ 564.0038 Found 564.0033.

3.5.7.(E)-2-(2-(2-((1H-benzo[d]imidazol-2-yl)thio)-1-(4-nitrophenyl)ethylidene)hydrazinyl)-4-(3-bromophenyl)thiazole (7)

Yield: (76%); mp. 209-208 °C;^{1}HNMR: (500 MHz, DMSO-d_6) δ 12.55 (s, 1H, -NHbi), 11.98 (s, 1H, -NH), 8.35(d, $J_{(3, 2)/(5, 6)}$ = 7.8Hz, 2H, H-3/H-5, ArH), 8.07 (d, $J_{(2, 3)/(6, 5)}$ = 7.6Hz, 2H, H-2/H-6,ArH), 7.82 (m, 1H, Ar'H), 7.66 (m, 1H, Ar'H), 7.44-7.46 (m, 2H, Ar'H), 7.43 (m, 2H, bi-H), 7.28 (s, 1H, thiazole-H), 7.12 (m, 2H, bi-H), 3.78 (s, 2H, -CH_2) ^{13}CNMR (125 MHz, DMSO-d_6): δ171.7, 155.6, 150.2, 150.2, 147.1, 140.1, 138.9, 138.9, 135.2, 131.4, 131.0, 128.1, 127.7, 127.7, 127.0, 127.0, 126.5, 123.0, 123.0, 122.2, 115.2, 115.2, 105.0, 37.5: HREI-MS: m/z calcld for $C_{24}H_{17}BrN_6O_2S_2$ $[M]^+$ 564.0038 Found 564.0033.

3.5.8.(E)-2-(2-(2-((1H-benzo[d]imidazol-2-yl)thio)-1-(4-nitrophenyl)ethylidene)hydrazinyl)-4-(2-bromophenyl)thiazole (8)

Yield: (75%); mp. 208-207 °C;^{1}HNMR: (500 MHz, DMSO-d_6) δ 12.55 (s, 1H, -NHbi), 11.98 (s, 1H, -NH), 8.35(d, $J_{(3, 2)/(5, 6)}$ = 7.8Hz, 2H, H-3/H-5, ArH), 8.07 (d, $J_{(2, 3)/(6, 5)}$ = 7.6Hz, 2H, H-2/H-6,ArH), 7.72-7.68 (m, 2H, Ar'H), 7.53 (m, 1H,Ar'H), 7.46 (m, 1H, Ar'H), 7.42 (m, 2H, bi-H), 7.12 (m, 2H, bi-H), 7.05 (s, 1H, thiazole-H), 3.78 (s, 2H, -CH_2) ^{13}CNMR (125 MHz, DMSO-d_6): δ171.7, 115.6, 150.2, 148.8, 147.1, 140.1, 139.8, 138.7, 138.7, 132.1, 130.9, 129.7, 128.2, 127.7, 127.7, 127.0, 127.0, 123.0, 123.0, 120.2, 115.2, 115.2, 105.0, 37.5: HREI-MS: m/z calcld for $C_{24}H_{16}Cl_2N_6O_2S_2$ $[M]^+$ 564.0038 Found 564.0033.

3.5.9.(E)-2-(2-(2-((1H-benzo[d]imidazol-2-yl)thio)-1-(4-nitrophenyl)ethylidene)hydrazinyl)-4-(4-nitrophenyl)thiazole (9)

Yield: (71%); mp. 205-206°C;^{1}HNMR: (500 MHz, DMSO-d_6) δ 12.55 (s, 1H, -NHbi), 11.98 (s, 1H, -NH), 8.35(d, $J_{(3, 2)/(5, 6)}$ = 7.8Hz, 2H, H-3/H-5, ArH), 8.28 (d, $J_{(3', 2')/(5', 6')}$ = 7.8Hz, 2H, H-3/H-5,Ar'H), 7.76(d, $J_{(2', 3')/(6', 5')}$ = 7.9Hz, 2H, H-2/H-6, Ar'H), 8.07 (d, $J_{(2, 3)/(6, 5)}$ = 7.6Hz, 2H, H-2/H-6,ArH), 7.43 (m, 2H, bi-H), 7.28 (s, 1H, thiazole-H), 7.12 (m, 2H, bi-H), 3.76 (s, 2H, -CH_2) ^{13}CNMR (125 MHz, DMSO-d_6); δ171.7, 155.6, 150.2, 150.2, 147.9, 147.1, 140.1, 138.9, 138.9, 127.7, 127.7, 127.0, 127.0, 126.2, 126.2, 124.4, 124.4, 123.0, 123.0, 115.2, 115.2, 105.0, 37.5: HREI-MS: m/z calcld for $C_{24}H_{17}N_7O_4S_2$ $[M]^+$ 531.0783 Found 531.0777.

3.5.10.(E)-2-(2-(2-((1H-benzo[d]imidazol-2-yl)thio)-1-(4-nitrophenyl)ethylidene) hydrazinyl)-4-(2-nitrophenyl)thiazole (10)

Yield: (69%); mp. 204-205°C;^{1}HNMR: (500 MHz, DMSO-d_6) δ 12.55 (s, 1H, -NHbi), 11.98 (s, 1H, -NH), 8.35(d, $J_{(3, 2)/(5, 6)}$ = 7.8Hz, 2H, H-3/H-5, ArH), 8.09 (d, $J_{(2, 3)/(6, 5)}$ = 7.6Hz, 2H, H-2/H-6,ArH), 8.01-8.03 (m, 2H, Ar'H), 7.90 (m, 1H,Ar'H), 7.72 (m, 1H, Ar'H), 7.42 (m, 2H, bi-H), 7.12 (m, 2H, bi-H), 7.05 (s, 1H, thiazole-H), 3.78 (s, 2H, -CH_2) ^{13}CNMR (125 MHz, DMSO-d_6): δ171.7, 155.6, 150.2, 148.8, 147.8, 147.1, 140.1, 138.9, 138.9, 135.3, 132.6, 129.6, 127.7, 127.7, 127.0, 127.0, 125.2, 124.4, 123.0, 123.0, 115.2, 115.2, 105.0, 37.5: HREI-MS: m/z calcld for $C_{24}H_{16}Cl_2N_6O_2S_2$ $[M]^+$ 531.0783 Found 531.0777.

3.5.11.(E)-2-(2-(2-((1H-benzo[d]imidazol-2-yl)thio)-1-(4-nitrophenyl)ethylidene) hydrazinyl)-4-(p-tolyl)thiazole (11)

Yield: (73%); mp. 198-197 °C;^{1}HNMR: (500 MHz, DMSO-d_6) δ 12.55 (s, 1H, -NHbi), 11.98 (s, 1H, -NH), 8.35(d, $J_{(3, 2)/(5, 6)}$ = 7.8Hz, 2H, H-3/H-5, ArH), 8.07 (d, $J_{(2, 3)/(6, 5)}$ = 7.6Hz, 2H, H-2/H-6,ArH), 7.86(d, $J_{(2', 3')/(6', 5')}$ = 7.9Hz, 2H, H-2/H-6, Ar'H), 7.43 (m, 2H, bi-H), 7.28 (s, 1H, thiazole-H), 7.26 (d, $J_{(3', 2')/(5', 6')}$ = 7.8Hz, 2H, H-3/H-5,Ar'H), 7.12 (m, 2H, bi-H), 3.76 (s, 2H, -CH_2), 2.35 (s, 1H, -CH_3), ^{13}CNMR (125 MHz, DMSO-d_6): δ171.7, 155.6, 150.2, 148.8, 147.8, 147.1, 140.1, 138.9, 138.9, 135.3, 132.6, 129.6, 127.7, 127.7, 127.0, 127.0, 125.2, 124.4, 123.0, 123.0, 115.2, 115.2, 105.0, 37.5: HREI-MS: m/z calcld for $C_{25}H_{20}N_6O_2S_2$ $[M]^+$ 500.1089 Found 500.1084.

3.5.12.(E)-2-(2-(2-((1H-benzo[d]imidazol-2-yl)thio)-1-(4-nitrophenyl)ethylidene) hydrazinyl)-4-(m-tolyl)thiazole (12)

Yield: (78%); mp. 197-196 °C;^{1}HNMR: (500 MHz, DMSO-d_6) δ 12.55 (s, 1H, -NHbi), 11.98 (s, 1H, -NH), 8.35(d, $J_{(3, 2)/(5, 6)}$ = 7.8Hz, 2H, H-3/H-5, ArH), 8.07 (d, $J_{(2, 3)/(6, 5)}$ = 7.6Hz, 2H, H-2/H-6,ArH), 7.82 (t, J=6.4Hz, 1H, Ar'H), 7.75 (m, 1H, Ar'H), 7.51 (m, 1H, Ar'H), 7.43 (m, 2H, bi-H), 7.28 (s, 1H, thiazole-H), 7.12 (m, 2H, bi-H), 7.18 (m, 1H, Ar'H), 3.78 (s, 2H, -CH_2) 2.45 (s, 1H, -CH_3), ^{13}CNMR (125 MHz, DMSO-d_6): δ171.7, 155.6, 150.2, 150.2, 147.1, 140.1, 138.9, 138.9, 138.9, 132.9, 130.4, 129.1, 129.0, 127.7, 127.7, 127.0, 127.0, 124.5, 123.0, 123.0, 115.2, 115.2, 105.0, 37.5, 21.6: HREI-MS: m/z calcld for $C_{24}H_{16}Cl_2N_6O_2S_2$ $[M]^+$ 500.1089 Found 500.1084.

3.5.13.(E)-2-(2-(2-((1H-benzo[d]imidazol-2-yl)thio)-1-(4-nitrophenyl)ethylidene) hydrazinyl)-4-(o-tolyl)thiazole (13)

Yield: (72%); mp. 196-195°C;^{1}HNMR: (500 MHz, DMSO-d_6) δ 12.55 (s, 1H, -NHbi), 11.98 (s, 1H, -NH), 8.35(d, $J_{(3, 2)/(5, 6)}$ = 7.8Hz, 2H, H-3/H-5, ArH), 8.09 (d, $J_{(2, 3)/(6, 5)}$ = 7.6Hz, 2H, H-2/H-6,ArH), 7.59 (m, 1H, Ar'H), 7.42 (m, 2H, bi-H), 7.36 (m, 2H,Ar'H), 7.26 (m, 1H, Ar'H), 7.12 (m, 2H, bi-H), 7.05 (s, 1H, thiazole-H), 3.78 (s, 2H, -CH_2),2.55 (s, 1H, -CH_3), ^{13}CNMR (125 MHz, DMSO-d_6): δ171.7, 155.6, 150.2, 147.8, 147.1, 140.1, 138.9, 138.9, 136.9, 129.7, 129.5, 128.6, 127.7, 127.7, 127.0, 127.0, 126.2, 123.0, 123.0, 122.7, 115.2, 115.2, 105.0, 37.5, 18.7: HREI-MS: m/z calcld for $C_{24}H_{16}Cl_2N_6O_2S_2$ $[M]^+$ 500.1089 Found 500.1084.

3.5.14.(E)-2-(2-(2-((1H-benzo[d]imidazol-2-yl)thio)-1-(4-nitrophenyl)ethylidene) hydrazinyl)-4-(4-chlorophenyl)thiazole (14)

Yield: (73%); mp. 204-205°C;^{1}HNMR: (500 MHz, DMSO-d_6) δ 12.55 (s, 1H, -NHbi), 11.98 (s, 1H, -NH), 8.35(d, $J_{(3, 2)/(5, 6)}$ = 7.8Hz, 2H, H-3/H-5, ArH), 8.07 (d, $J_{(2, 3)/(6, 5)}$ = 7.6Hz, 2H, H-2/H-6,ArH), 8.04(d, $J_{(2', 3')/(6', 5')}$ = 7.9Hz, 2H, H-2'/H-6', Ar'H), 7.56 (d, $J_{(3', 2')/(5', 6')}$ = 7.8Hz, 2H, H-3'/H-5',Ar'H), 7.43 (m, 2H, bi-H), 7.28 (s, 1H, thiazole-H), 7.12 (m, 2H, bi-H), 3.76 (s, 2H, -CH_2), ^{13}CNMR (125 MHz, DMSO-d_6): δ171.7, 155.6, 150.2, 150.2, 147.1, 140.1, 138.9, 138.9, 134.3, 131.1, 129.3, 129.3, 128.9, 128.9, 127.7, 127.7, 127.0, 127.0, 123.0, 123.0, 115.2, 115.2, 105.0, 37.5: HREI-MS: m/z calcld for $C_{24}H_{17}ClN_6O_2S_2$ $[M]^+$ 520.0543 Found 520.0538.

3.5.15.(E)-2-(2-(2-((1H-benzo[d]imidazol-2-yl)thio)-1-(4-nitrophenyl)ethylidene) hydrazinyl)-4-(2-chlorophenyl)thiazole (15)

Yield: (67%); mp. 204-203°C;^{1}HNMR: (500 MHz, DMSO-*d6*) δ 12.55 (s, 1H, -NHbi), 11.98 (s, 1H, -NH), 8.35(d, $J_{(3, 2)/(5, 6)}$ = 7.8Hz, 2H, H-3/H-5, ArH), 8.07 (d, $J_{(2, 3)/(6, 5)}$ = 7.6Hz, 2H, H-2/H-6,ArH), 7.71 (m, 1H, Ar'H), 7.60 (m, 1H,Ar'H), 7.42 (m, 2H, bi-H), 7.37 (m, 2H, Ar'H), 7.12 (m, 2H, bi-H), 7.05 (s, 1H, thiazole-H), 3.78 (s, 2H, -CH_2) ^{13}CNMR (125 MHz, DMSO-*d6*): δ171.7, 155.6, 150.2, 147.8, 147.1, 140.1, 138.9, 138.9, 132.5, 132.2, 130.6, 130.1, 129.9, 128.9, 127.7, 127.7, 127.0, 127.0, 123.0, 123.0, 115.2, 115.2, 105.0, 37.5: HREI-MS: m/z calcld for $C_{24}H_{16}Cl_2N_6O_2S_2$ $[M]^+$ 520.0543 Found 520.0538.

3.5.16.(E)-2-(2-(2-((1H-benzo[d]imidazol-2-yl)thio)-1-(4-nitrophenyl)ethylidene) hydrazinyl)-4-(3-chlorophenyl)thiazole (16)

Yield: (74%); mp. 203-202 °C;^{1}HNMR: (500 MHz, DMSO-*d6*) δ 12.55 (s, 1H, -NHbi), 11.98 (s, 1H, -NH), 8.35(d, $J_{(3, 2)/(5, 6)}$ = 7.8Hz, 2H, H-3/H-5, ArH), 8.07 (d, $J_{(2, 3)/(6, 5)}$ = 7.6Hz, 2H, H-2/H-6,ArH), 7.97 (t, *J*=6.3Hz, 1H, Ar'H), 7.72 (m, 1H, Ar'H), 7.49 (m, 2H, Ar'H), 7.43 (m, 2H, bi-H), 7.28 (s, 1H, thiazole-H), 7.12 (m, 2H, bi-H), 3.78 (s, 2H, -CH_2), ^{13}CNMR (125 MHz, DMSO-*d6*): δ171.7, 155.6, 150.2, 150.2, 147.1, 140.1, 138.9, 138.9, 134.8, 134.4, 129.6, 129.5, 128.8, 127.7, 127.7, 127.0, 127.0, 125.6, 123.0, 123.0, 115.2, 115.2, 105.0, 37.5: HREI-MS: m/z calcld for $C_{24}H_{16}Cl_2N_6O_2S_2$ $[M]^+$ 520.0543 Found 520.0538.

3.5.17.(E)-2-(2-(2-((1H-benzo[d]imidazol-2-yl)thio)-1-(4-nitrophenyl)ethylidene)hydrazinyl)-4-(3-nitrophenyl)thiazole (17)

Yield: (77%); mp. 201-200°C;^{1}HNMR: (500 MHz, DMSO-*d6*) δ 11.89 (s, 1H, NH), 11.77 (s, 1H, NH), 8.79 (d, $J_{(3, 2)/(5, 6)}$ = 7.7Hz, 2H, H-3/H-5, ArH), 8.51 (d, $J_{(2, 3)/(6, 5)}$ = 6.7Hz, 2H, H-2/H-6, ArH), 8.43 (s, 1H, Ar'H), 7.58 (s, 1H, thiazole-H), 7.57 (dd, *J* = 7.1, 2.4Hz, 1H, bi-H), 7.55-7.52 (m, 1H, bi-H), 7.51 (dd, *J* = 6.9, 2.0Hz, 1H, Ar'H), 7.40-7.38 (m, 1H, bi-H),7.35 (t, *J* = 6.3Hz, 1H, Ar'H), 7.25 (dd, *J*= 7.5, 1.9Hz, 1H, Ar'H), 6.61 (dd, *J* = 7.3, 1.5Hz, 1H, bi-H), 3.25 (s, 2H, -CH_2), ^{13}CNMR (125 MHz, DMSO-*d6*):δ 168.4, 167.0, 148.9, 146.7, 142.1, 141.5, 138.6, 137.9, 137.7, 136.9, 132.8, 132.7, 132.3, 132.0, 130.1, 128.9, 127.0, 126.1, 125.0, 120.6, 117.7, 110.3, 109.6, 40.0: HREI-MS: m/z calcld for $C_{24}H_{17}N_7O_4S_2$ $[M]^+$ 520.0783, Found, 520.0777.

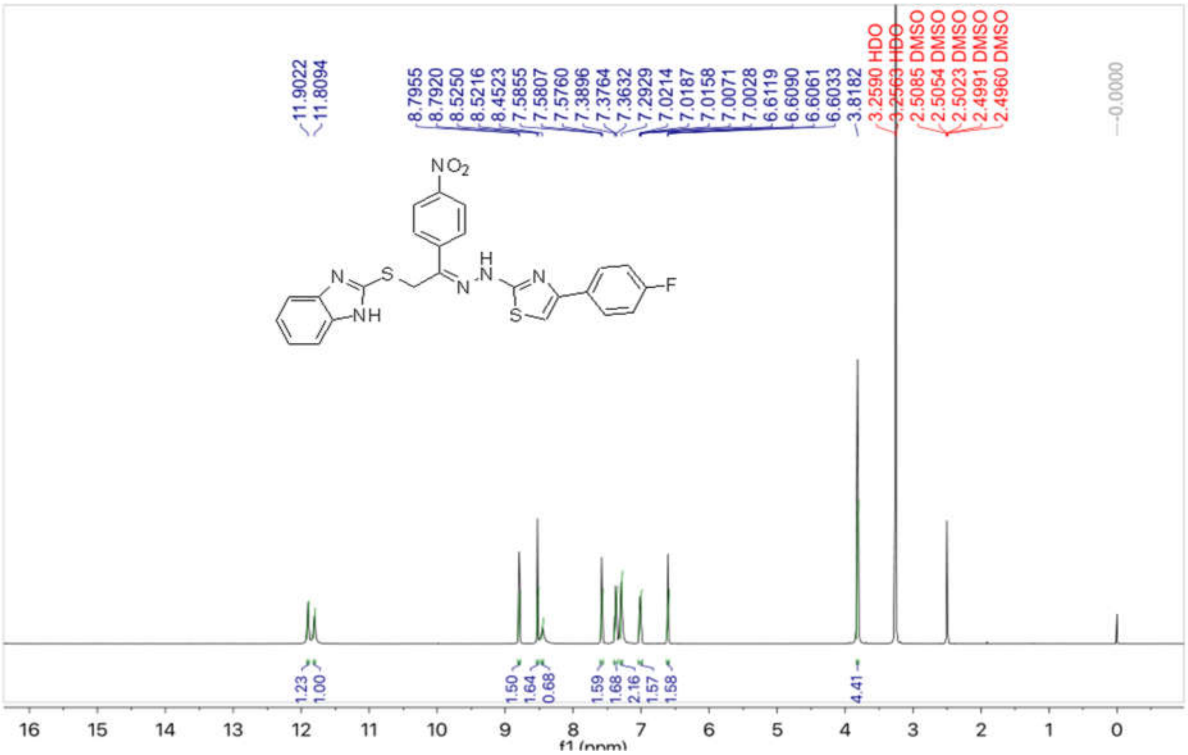

Figure S1. Low resolution Proton NMR Spectrum of*(E)-2-(2-(2-((1H-benzo[d]imidazol-2-yl)thio)-1-(4-nitrophenyl)ethylidene)hydrazinyl)-4-(4-fluorophenyl)thiazole (3)*

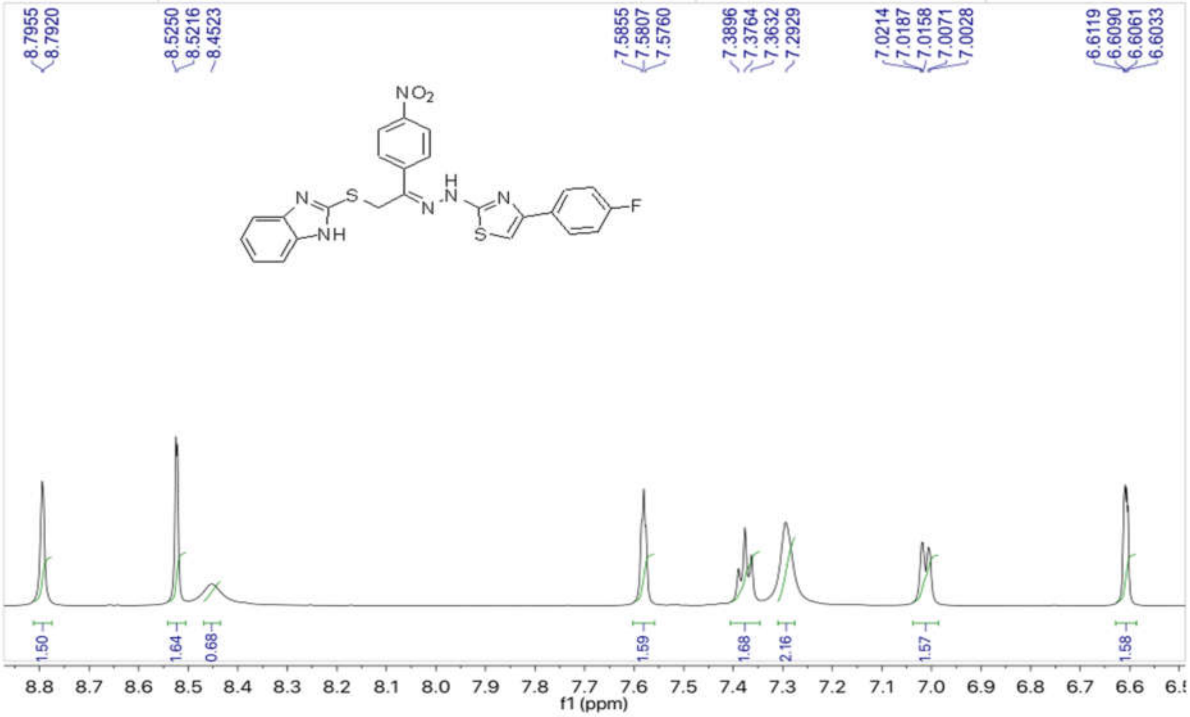

Figure S2. High resolution Proton NMR Spectrum of *(E)-2-(2-(2-((1H-benzo[d]imidazol-2-yl)thio)-1-(4-nitrophenyl)ethylidene)hydrazinyl)-4-(4-fluorophenyl)thiazole (3)*

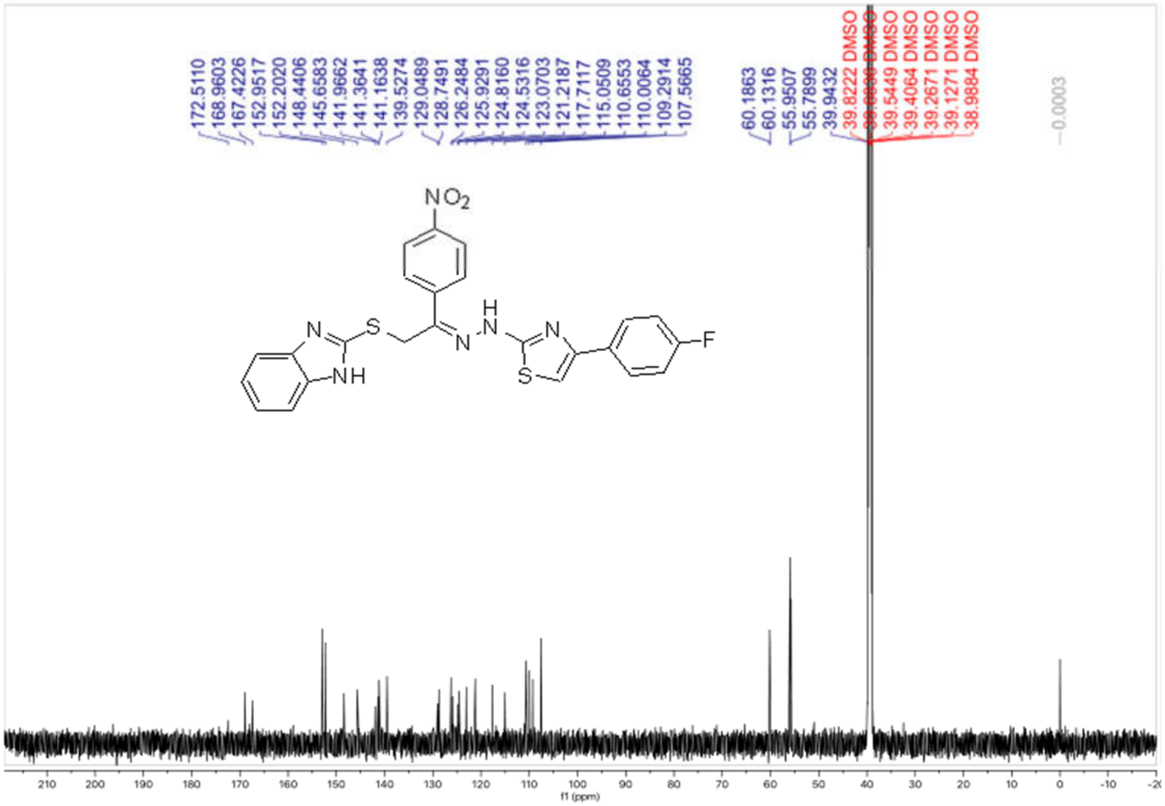

Figure S3. ^{13}C-NMR Spectrum of *(E)-2-(2-(2-((1H-benzo[d]imidazol-2-yl)thio)-1-(4-nitrophenyl)ethylidene)hydrazinyl)-4-(4-fluorophenyl)thiazole (3)*

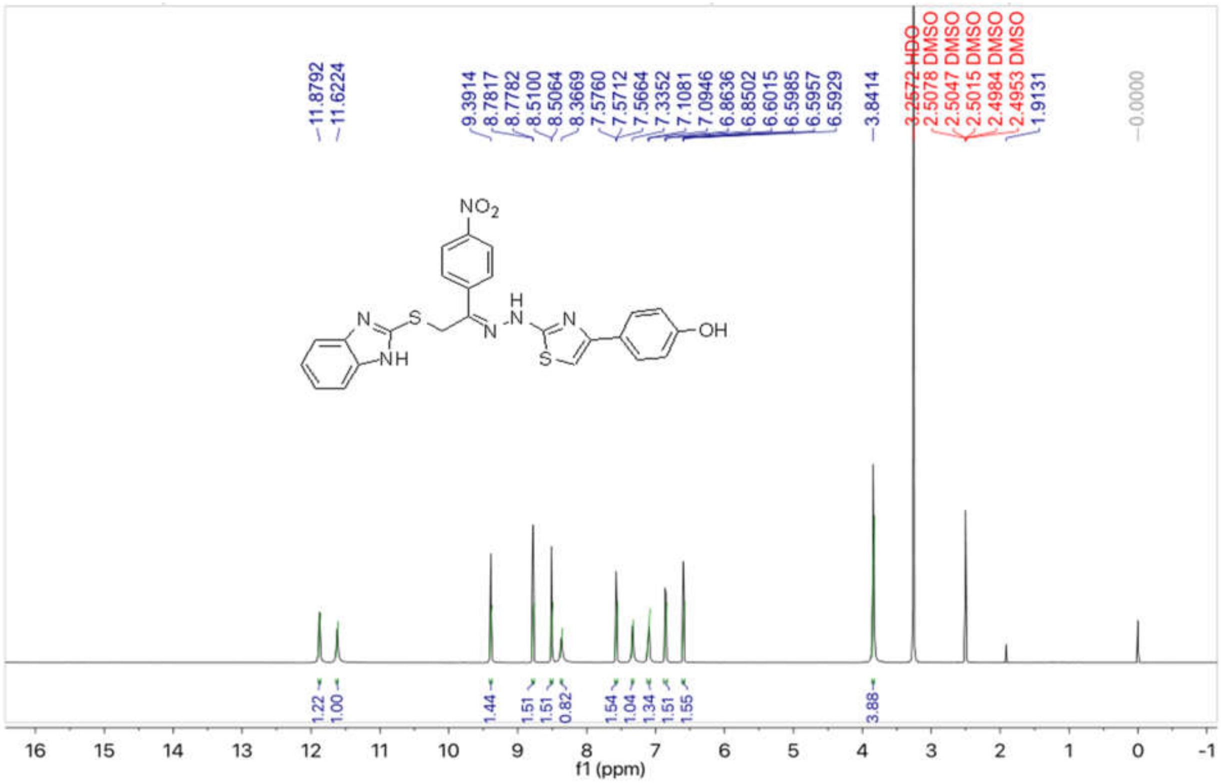

Figure S4. Low resolution Proton NMR Spectrum of *(E)-4-(2-(2-(2-((1H-benzo[d]imidazol-2-yl)thio)-1-(4-nitrophenyl)ethylidene)hydrazinyl) thiazol-4-yl)phenol (5)*

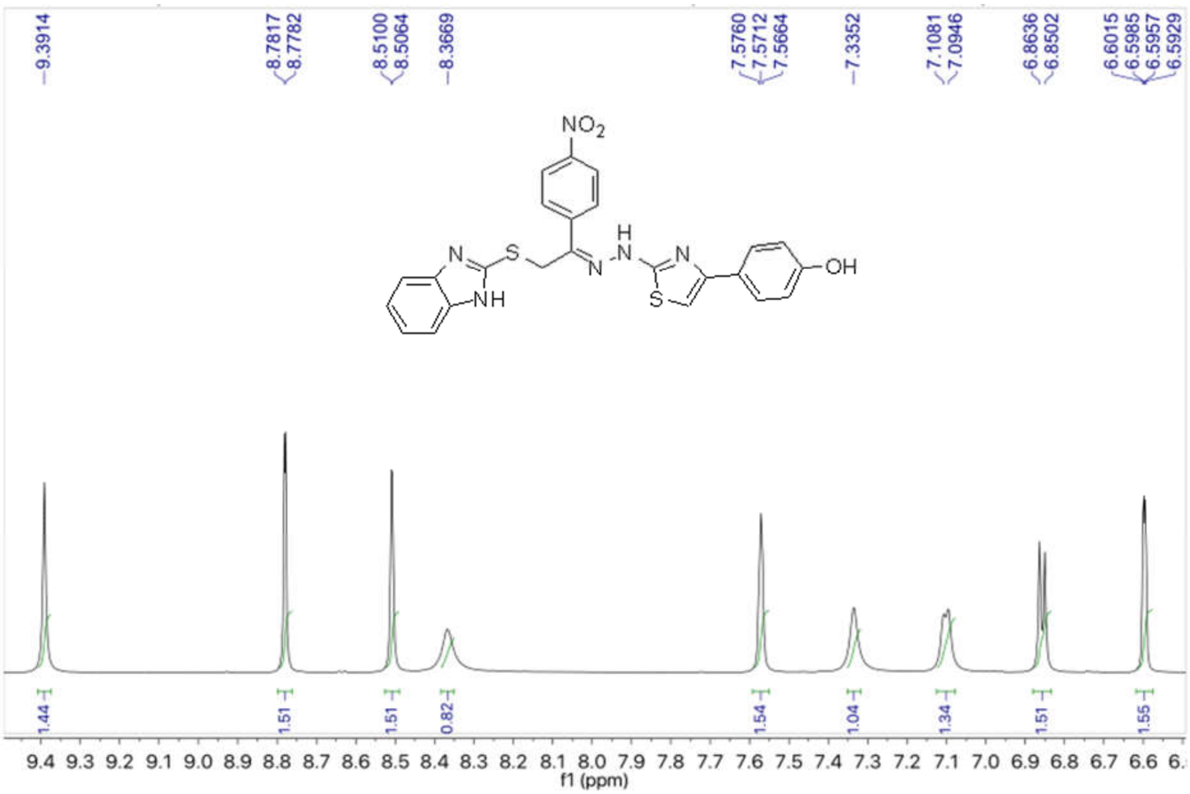

Figure S5. High resolution Proton NMR Spectrum of *(E)-4-(2-(2-(2-((1H-benzo[d]imidazol-2-yl)thio)-1-(4-nitrophenyl)ethylidene)hydrazinyl) thiazol-4-yl)phenol (5)*

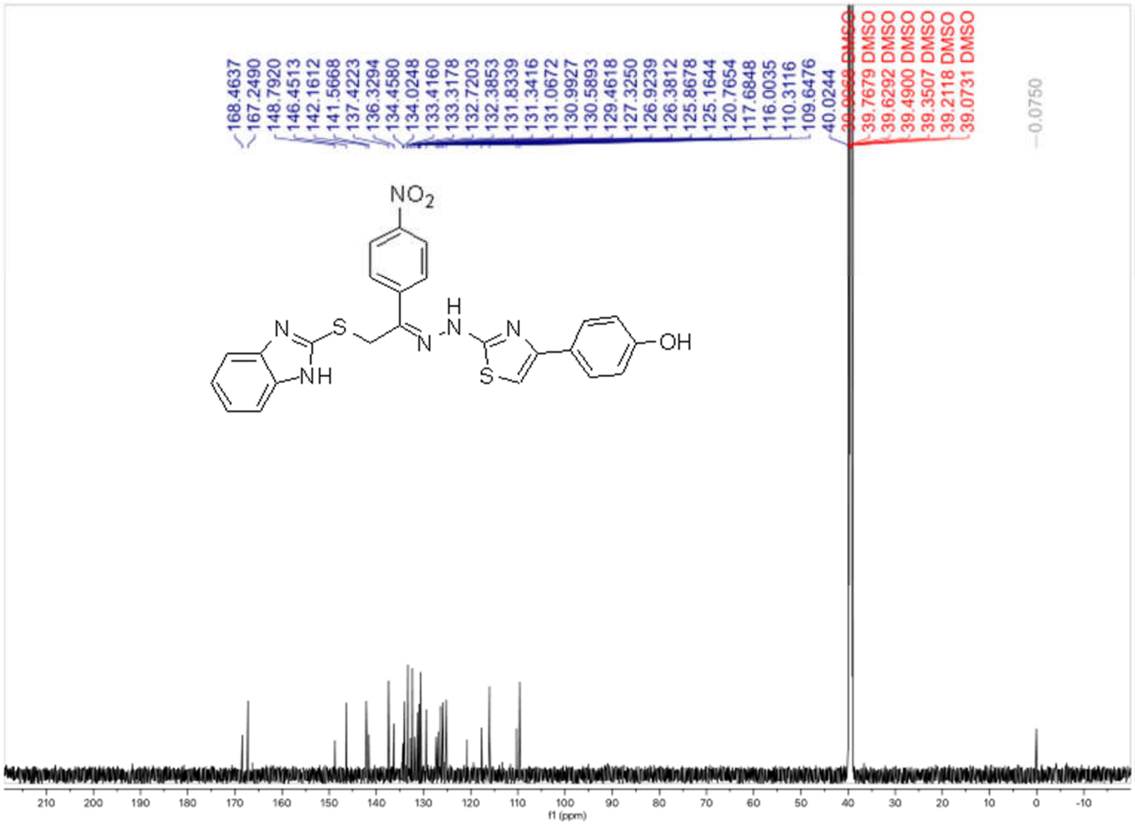

Figure S6.^{13}C-NMR Spectrum of *(E)-4-(2-(2-(2-((1H-benzo[d]imidazol-2-yl)thio)-1-(4-nitrophenyl)ethylidene)hydrazinyl) thiazol-4-yl)phenol (5)*

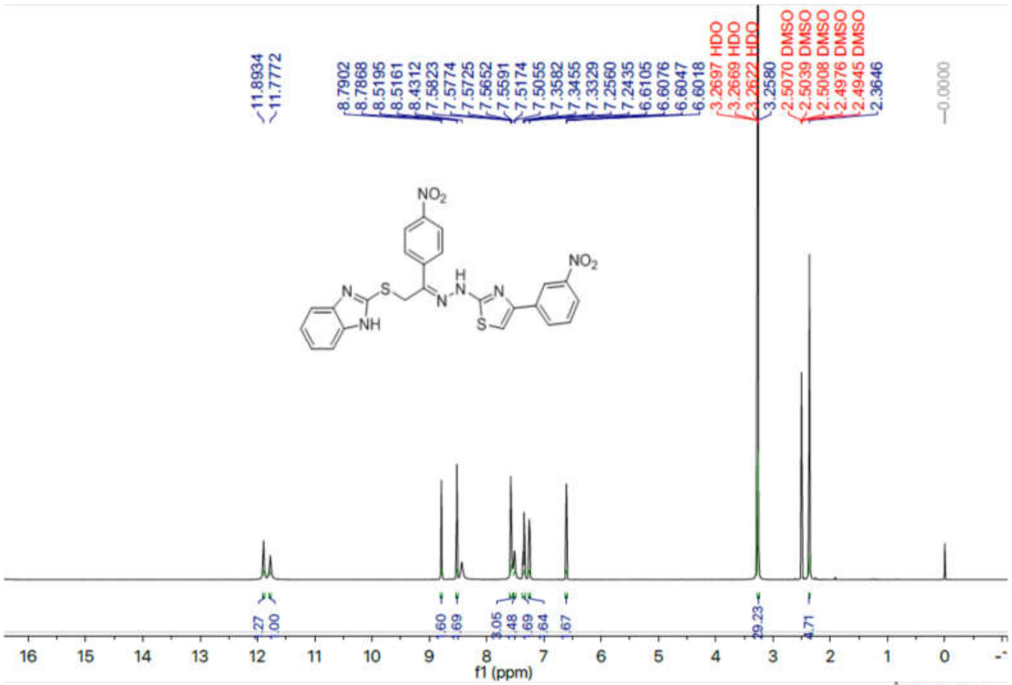

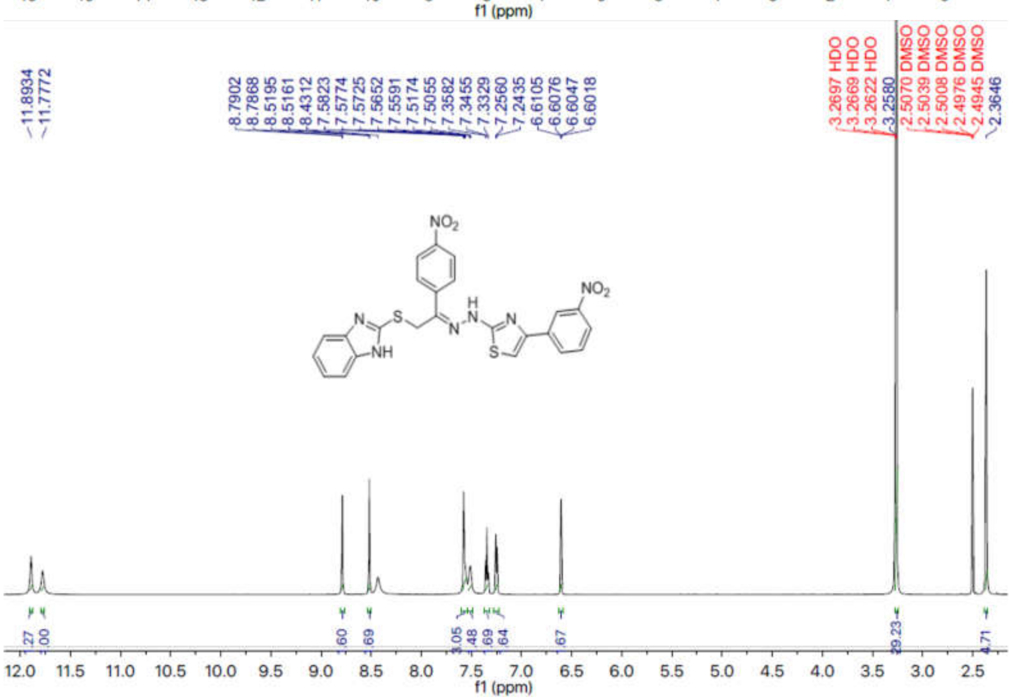

Figure S7. Low resolution proton NMR Spectrum of *(E)-2-(2-(2-((1H-benzo[d]imidazol-2-yl)thio)-1-(4-nitrophenyl)ethylidene)hydrazinyl)-4-(3-nitrophenyl)thiazole (17)*

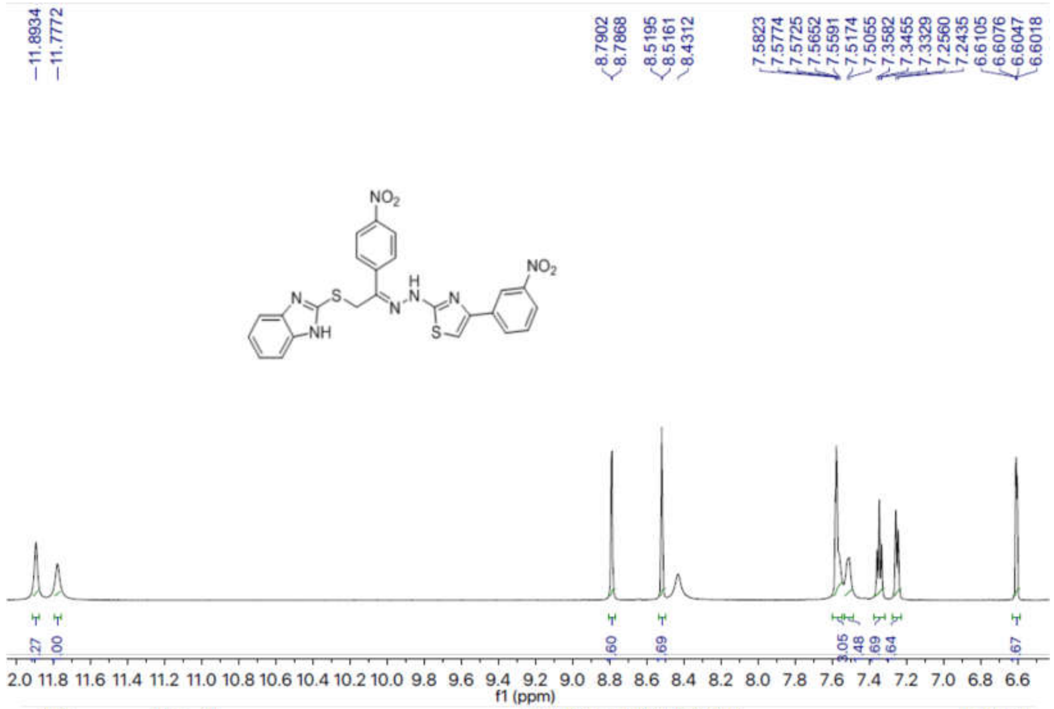

11.8934
11.7772
8.7902
8.7868
8.5195
8.5161
8.4312
7.5823
7.5774
7.5725
7.5652
7.5591
7.5174
7.5055
7.3582
7.3455
7.3329
7.2560
7.2435
6.6105
6.6076
6.6047
6.6018
NO2
N
S
NH
H
N
N
N
S
NO2
f1 (ppm)

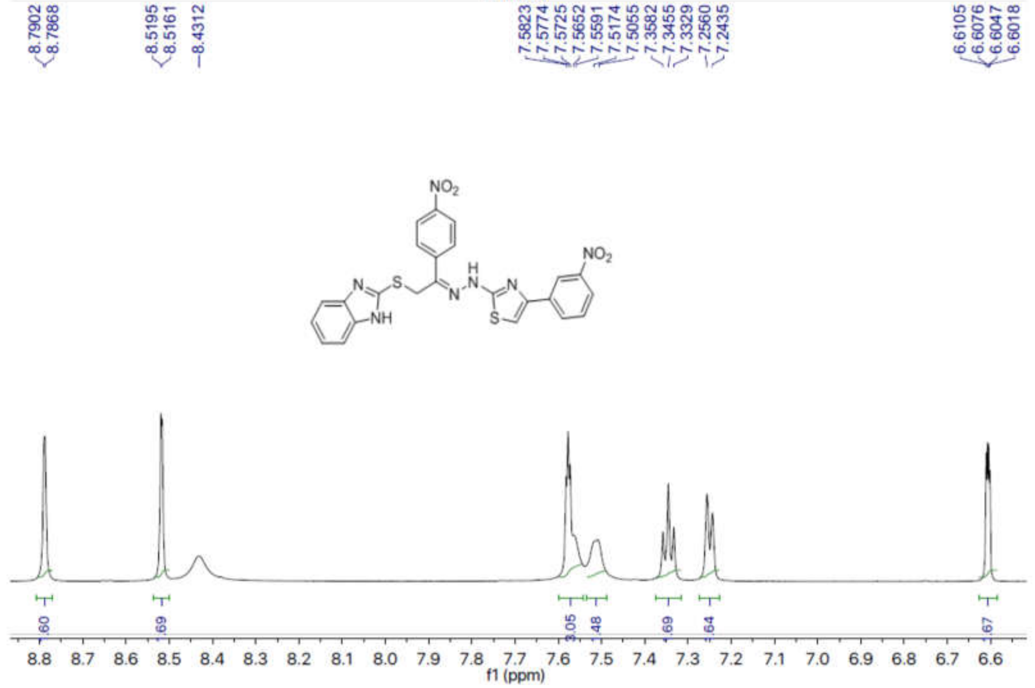

8.7902
8.7868
8.5195
8.5161
8.4312
7.5823
7.5774
7.5725
7.5652
7.5591
7.5174
7.5055
7.3582
7.3455
7.3329
7.2560
7.2435
6.6105
6.6076
6.6047
6.6018
NO2
N
S
NH
H
N
N
N
S
NO2
f1 (ppm)

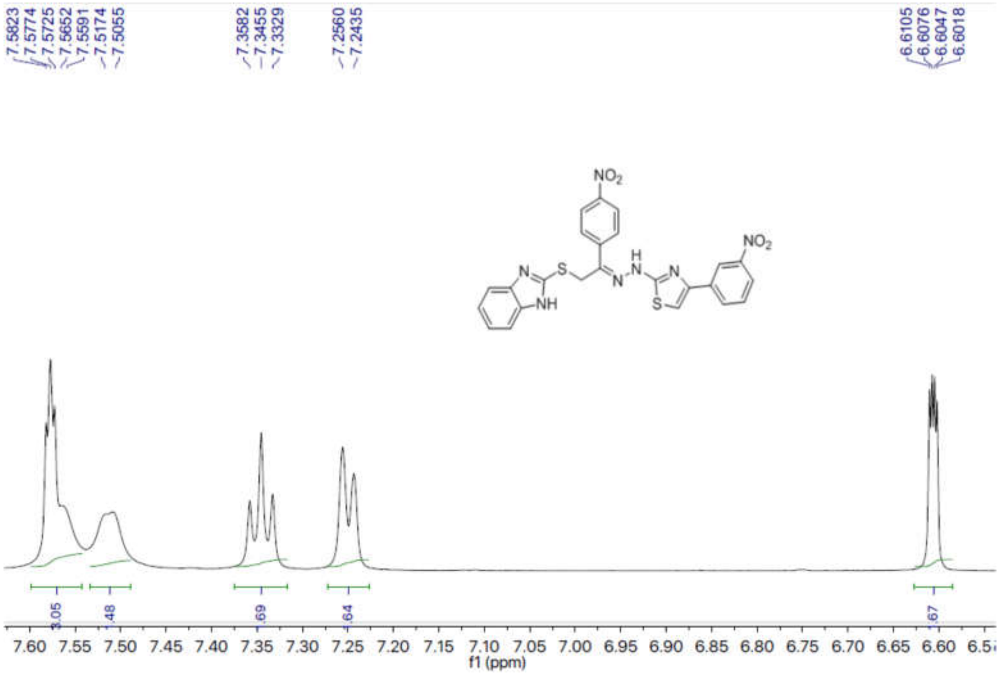

Figure S8. High resolution proton NMR Spectrum of *(E)-2-(2-(2-((1H-benzo[d]imidazol-2-yl)thio)-1-(4-nitrophenyl)ethylidene)hydrazinyl)-4-(3-nitrophenyl)thiazole (17)*

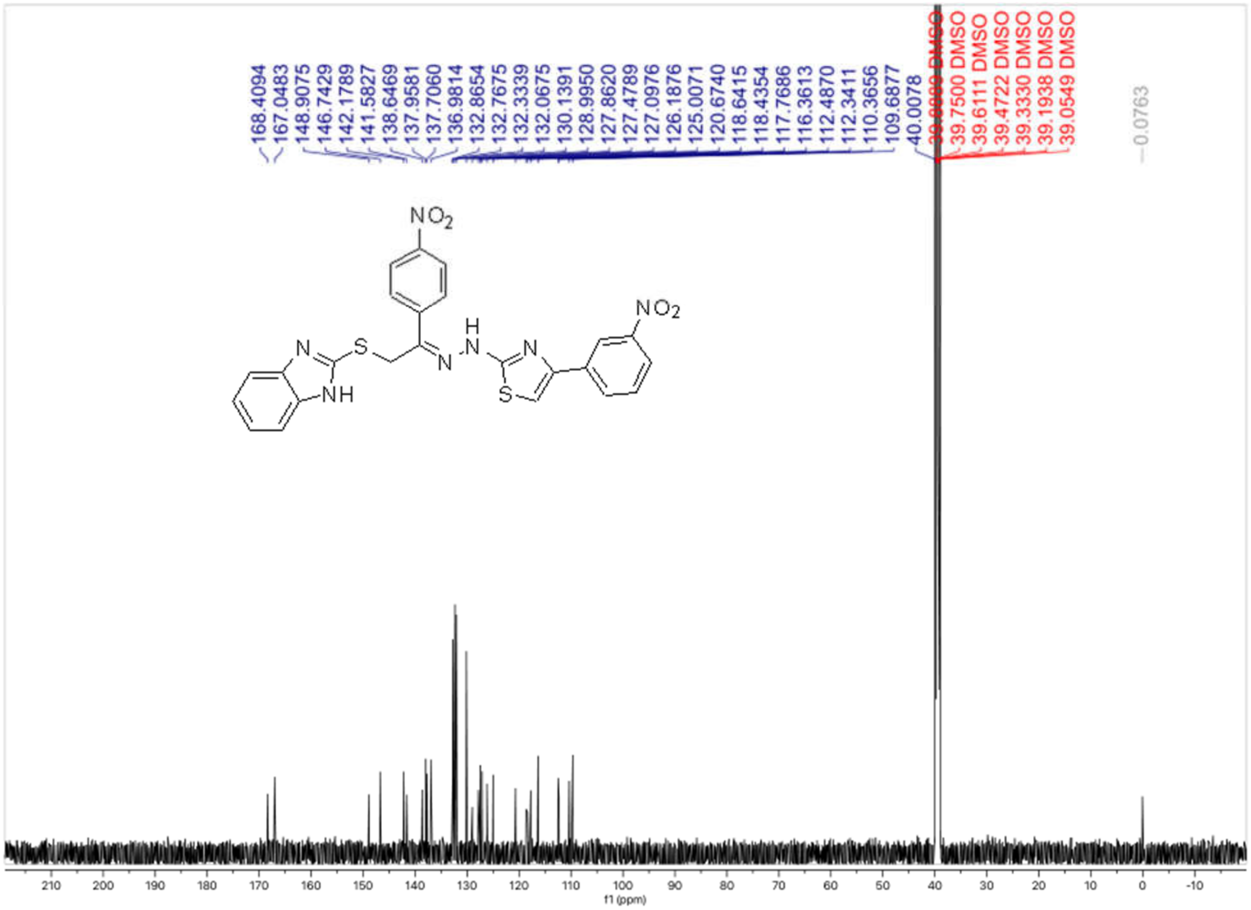

Figure S9. Low resolution ^{13}C-NMR Spectrum of *(E)-2-(2-(2-((1H-benzo[d]imidazol-2-yl)thio)-1-(4-nitrophenyl)ethylidene)hydrazinyl)-4-(3-nitrophenyl)thiazole (17)*

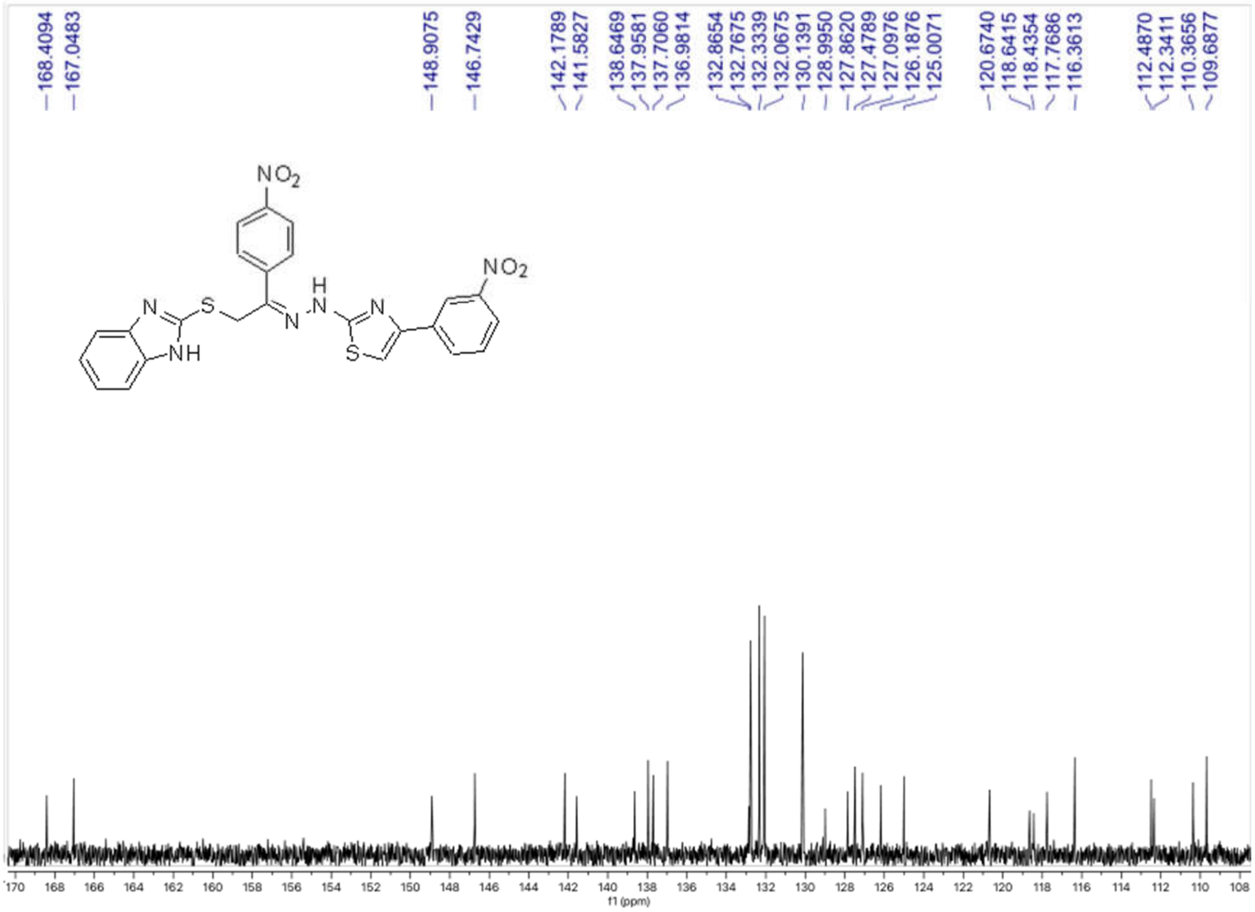

Figure S10. High resolution ^{13}C-NMR Spectrum of *(E)-2-(2-(2-((1H-benzo[d]imidazol-2-yl)thio)-1-(4-nitrophenyl)ethylidene)hydrazinyl)-4-(3-nitrophenyl)thiazole (17)*

1. Blum, C.; Roli, A.; Sampels, M. (Eds.) *Hybrid Metaheuristics: An Emerging Approach to Optimization*; Springer: Berlin, Germany, **2008;** Volume 114.
2. Baxter, J. Local optima avoidance in depot location. *J. Oper. Res. Soc.* **1981**, *32*, 815–819.
3. Azam, S.S.; Abbasi, S.W. Molecular docking studies for the identification of novel melatoninergic inhibitors for acetylserotonin-O-methyltransferase using different docking routines. *Theor. Biol. Med. Model.* **2013**, *10*, 63.
